# Musical hallucinations, musical imagery, and earworms: A new phenomenological survey

**DOI:** 10.1016/j.concog.2018.07.009

**Published:** 2018-10

**Authors:** Peter Moseley, Ben Alderson-Day, Sukhbinder Kumar, Charles Fernyhough

**Affiliations:** a Psychology Department, Durham University, South Road, Durham DH1 3LE, United Kingdom; b School of Psychology, University of Central Lancashire, Marsh Lane, Preston PR1 2HE, United Kingdom; c Institute of Neuroscience, Newcastle University, Newcastle NE1 7RU, United Kingdom

**Keywords:** Auditory hallucinations, Musical hallucinations, Earworms, Mental imagery, Phenomenology

## Abstract

Musical hallucinations (MH) account for a significant proportion of auditory hallucinations, but there is a relative lack of research into their phenomenology. In contrast, much research has focused on other forms of internally generated musical experience, such as earworms (involuntary and repetitive inner music), showing that they can vary in perceived control, repetitiveness, and in their effect on mood. We conducted a large online survey (*N* = 270), including 44 participants with MH, asking participants to rate imagery, earworms, or MH on several variables. MH were reported as occurring less frequently, with less controllability, less lyrical content, and lower familiarity, than other forms of inner music. MH were also less likely to be reported by participants with higher levels of musical expertise. The findings are outlined in relation to other forms of hallucinatory experience and inner music, and their implications for psychological models of hallucinations discussed.

## Highlights

• We conducted a phenomenological survey comparing musical hallucinations to other inner music.
• MH were more likely to be reported as externally located than other experiences.
• MH were less controllable and less familiar than imagery or earworms.
• MH were less likely to include lyrical content than other forms of inner music.
• Individuals with higher levels of musical expertise were less likely to report MH.

## 1 Introduction

Auditory hallucinations (AH) are defined as the conscious experience of sounds that occur in the absence of any actual sensory input. Although the most frequently reported form of AH are auditory verbal hallucinations (AVH), phenomenological surveys have also shown that a substantial minority of people also report musical hallucinations (MH): that is, the perception of music when none is playing. For example, one survey of 100 people with psychosis and AVH found that 36% also described the occurrence of MH (Nayani & David, 1996). The most frequent reports were of hearing choral music, with orchestral music and pop music also evidenced, although specific frequencies were not provided. More recently, McCarthy-Jones et al. (2012) analyzed data from a semi-structured interview with 199 psychotic patients who reported AVH, finding that a smaller proportion (compared to Nayani and David) of approximately 15% also experienced MH. The two largest phenomenological surveys of AVH, then, suggest that MH occur in a substantial minority of people who hear voices; however, since both primarily focus on AVH, few details of MH are described beyond prevalence. Whilst questionnaire measures used to assess proneness to hallucinations (e.g., Launay-Slade Hallucination Scale; Morrison, Wells, & Nothard, 2000) in the general population do include items relating to non-verbal hallucinations, responses to individual items are rarely reported; thus, we know little about either the prevalence or phenomenology of MH in clinical or non-clinical samples. Indeed, non-verbal hallucinations have been somewhat neglected in the psychological literature, with only a small number of studies investigating risk factors and basic phenomenological features of MH.

Surveys focusing exclusively on MH have suggested that they may occur in around 16% of individuals with a diagnosis of schizophrenia (Saba & Keshavan, 1997), and as many as 41% of individuals with obsessive compulsive disorder (Hermesh et al., 2004). Other risk factors include hearing impairments, old age, and social isolation, although these may not be independent factors (Evers & Ellger, 2004). Few surveys have specifically investigated the phenomenology of MH, further than reporting the most frequent styles of music. Saba and Keshavan did report on several details of MH in a small sample of individuals with a diagnosis of schizophrenia, showing that the majority included both instrumental and lyrical elements, which tended to be familiar to the individual. Patients tended to appraise the MH fairly positively, with the most frequent description of the experience being ‘soothing’ (62%). Many experiences of MH were described as perceived as emanating from the external environment, and approximately half were described as outside of volitional control, which Saba and Keshavan argue should be considered a key feature of MH. Whilst this study provided important information on the experience of MH in schizophrenia, the sample size (16 participants reporting MH) was low, and the questions on phenomenology relatively limited.

Golden and Josephs (2015) recently reviewed medical records of individuals, including 393 cases of MH, grouping the data into five categories: MH associated with neurological disorder, psychiatric disorder, structural brain damage, drug toxicity, and those not otherwise classifiable. The study mainly reports on brain regions associated with MH, but does note that many individuals with psychiatric disorders found that the experiences were ‘mood-congruent’ (e.g., sad music when they were feeling depressed). Indeed, within psychiatric patients reporting MH, depression seems to be the most common diagnosis (69%), along with hearing loss or tinnitus (Golden and Josephs, 2015, Rocha et al., 2015, Teunisse and Olde-Rikkert, 2012). A case series presented by Warner and Aziz (2005) of patients referred to old-age psychiatric services, though, only found a rate of hearing loss of 33% in patients with MH – perhaps surprisingly low given a mean age of 78 years. They also note that many patients were not distressed by the MH, and so speculate that the prevalence of such phenomena may be higher than previously thought if individuals do not seek medical attention. Due to the nature of these studies, however, no participants from non-clinical populations were included. Other studies have also used stringent inclusion criteria: for example, Evers and Ellger, in a review of the etiology of MH, deliberately excluded musical ‘pseudohallucinations’ (those experienced as internal to the individual). The distinction between ‘true’ hallucinations and pseudohallucinations is no longer thought to be clinically significant (Copolov, Trauer, & Mackinnon, 2004), and research into AVHs typically includes both internally and externally located perceptions (Nayani & David, 1996). It is unclear to what extent MH are experienced as internal or external, but it is possible that previous research has omitted a significant number of cases by using overly strict inclusion criteria.

Furthermore, it is unclear to what extent the attributes assessed in the small amount of previous research could also be applied to other forms of ‘inner music’^1^ (Fernyhough, 2016, p. 238). Musical imagery, for example, is the generation of music in one’s own head, not necessarily instigated by any external percept. It is frequently reported by many individuals in the general population (Bailes, 2007, Williamson et al., 2012), and often used by musicians to rehearse or aid reproduction of music, in the form of notational audiation (Brodsky, Henik, Rubinstein, & Zorman, 2003). Musical imagery can also occur involuntarily (INMI) with little or no volitional control. One form of INMI, ‘earworms’ (also referred to as ‘sticky tunes’ or ‘stuck songs’), are typically defined by their repetitiveness and persistence (although there is some debate in the literature regarding how to precisely define the experience – see below). Previous research has indicated that the frequency of earworms is affected by exposure to, and rehearsal of, music (Liikkanen, 2012), and as such is elevated in musically trained individuals (Beaty et al., 2013, Floridou et al., 2015), with one experience sampling study in musicians finding musical imagery occurring in as many as 32% of randomly sampled episodes, with 58% of these samples noted as being due to having recently heard or rehearsed music (Bailes, 2007, Bailes, 2006). Earworms tend not to be associated with negative emotions, unless the reported duration is particularly lengthy (presumably due to unwanted persistence) (Floridou et al., 2015).

To our knowledge, no research has directly compared self-reported experiences of musical imagery and earworms to MH, and, in fact, the boundary between earworms and MH is somewhat unclear in much of the literature. For example, Hemming (cited in Williams, 2015) defines MH as INMI that reaches a pathological level (presumably reflected in distress experienced by the individual), implying that MH are simply a more extreme, persistent, or distressing version of earworms. In support of this, the aforementioned study by Saba and Keshavan (1997) distinguished MH from musical imagery purely in terms of volitional control. In contrast, Williams argues that whilst both MH and earworms are involuntary, only MH are experienced as located in the external environment. However, as discussed above, other forms of auditory hallucination, for example AVH, are often experienced as internally located (Daalman et al., 2011, Nayani and David, 1996), yet are typically still classified as hallucinatory experiences. An open question, then, is the extent to which earworms and MH share phenomenological attributes (e.g., control, perceived location), and whether MH can be distinguished on other aspects of musical experience (e.g., type of music, frequency, duration, familiarity, level of acoustic detail).

Research into musical imagery and earworms has also investigated their effect on mood and behavior, but again, these have not been directly investigated in comparison to MH. For example, Williamson, Liikkanen, Jakubowski, and Stewart (2014) showed that 74.6% of individuals reported humming or singing along in response to earworms, whilst only 10.9% reported attempting to suppress them. Participants in other studies have also reported that bodily movements in response to earworms (e.g., tapping a foot to the beat) are relatively common (Floridou et al., 2015). Finally, frequency of earworms was associated with self-reported obsessive-compulsive traits, perhaps similarly to the persistence of intrusive thoughts in obsessive-compulsive disorder (Beaman & Williams, 2010). In contrast, little is known about typical affective and behavioral responses to MH.

There are, then, several large gaps in the MH literature, which we sought to address in the present study. Firstly, very little research has been conducted regarding the phenomenology of MH much further than asking about the broad style of music experienced. Secondly, there is some confusion within the literature over precise definitions as to what constitutes an MH, leading previous studies to use different inclusion criteria. As mentioned, Saba and Keshavan (1997) used ‘volitional control’ as a key indicator of MH; yet, this alone fails to distinguish the experience from that of earworms. On the other hand, some authors seem to have equated earworms and MH (Hermesh et al., 2004), whilst others have equated earworms and INMI as referring to the same phenomenon (Farrugia, Jakubowski, Cusack, & Stewart, 2015). Williams (2015) has argued that INMI should be used as a broader term, defining any type of musical imagery outside of conscious control, with the term ‘earworm’ being restricted to a type of INMI characterized by its repetitiveness. Within Williams' framework, MH would be categorized as a form of INMI defined by their perceived externality and pathology (e.g. hearing impairment and/or brain damage). Williams, then, offers perhaps the most rigorous categorization of forms of inner music, but, due to a lack of previous research, does not discuss other potential differences between MH and earworms.

Supplementary data associated with this article can be found, in the online version, at https://doi.org/10.1016/j.concog.2018.07.009.

We sought to conduct an exploratory survey of the phenomenology of MH, to investigate potential similarities and differences between MH and other forms of inner music. Participants were asked to pick a category that they felt best described their inner musical experience (musical imagery, earworm, musical hallucination) based on basic definitions (see Supplementary Materials), or specify that they regularly experienced multiple different types of inner music (henceforth referred to as the ‘mixed experiences’ group). These categories were then compared on a number of phenomenological attributes, both based on those reported in previous research, and areas that have not previously been investigated. Based on previous literature, it was expected that MH would be more likely to be experienced as coming from the external environment, whereas earworms would be characterized by a lack of volition and repetitiveness, compared to musical imagery. As well as collecting demographic information, we also asked about the presence of psychiatric diagnoses and hearing impairments, as well as prior musical experience, given that previous literature suggests these may be key predictors of the presence of MH. Furthermore, we asked about a number of other features of the experience (frequency, duration, familiarity, feelings of anticipation, triggers), musical details (perception of lyrics, instruments, intensity, harmony, melody) and effects on behavior (effects on the body, effects on mood, effects on relationships with others). Participants were also given the chance to provide further information about their experiences in free text boxes on many questions. The aim was, therefore, to provide a more detailed and nuanced study of phenomenological aspects of MH as compared to other types of inner music, than has previously been conducted.

**Supplementary Material:** 

## 2 Materials and methods

### 2.1 Participants

Participants were invited to take part in an on-line survey, advertised via social media and a research project website (http://hearingthevoice.org/2014/11/04/round-and-round-the-phenomenology-of-inner-music). Rather than aiming to recruit a sample representative of the national population, the aim was to recruit participants who reported hallucinatory experiences or regular inner music, to investigate phenomenological features of these experiences.

There were 276 respondents to the questionnaire. From these, 7 participants were excluded who did not respond to a sufficient number of questions (<10% response rate) on the ‘Phenomenology of Inner Music Questionnaire’ (see below), whilst 14 that described an alternative form of inner music in the free text box (e.g., musical memory) were excluded. Thus, the sample analyzed consisted of 255 participants (105 male, 144 female, 6 other), with a mean age of 39.4 (*SD* = 13.3, range = 18–74). The majority of participants were from English-speaking countries (e.g., 42.4% USA, 34.1% UK) but there were also respondents from other European countries (e.g., Denmark, Germany, France, Finland) and non-European countries around the world (e.g., Israel, Mexico, South Korea). Some form of hearing loss was reported by 15.8% of participants, with most of these being described simply as hearing loss/hypacusis (80%) and/or tinnitus (37.5%). Mean reported length of hearing impairment in these individuals was 18.3 years (*SD* = 17.3). 42.7% of participants reported having previously received a psychiatric diagnosis, with the largest proportion of these being for depression (60.6%) or anxiety (30.3%), with smaller numbers of participants having diagnoses of bipolar disorder, obsessive compulsive disorder, attention deficit hyperactivity disorder, or schizophrenia/schizoaffective disorder/psychosis. 9.0% of participants reported some form of neurological disorder, whilst 26.4% reported being on some form of medication for a psychiatric/neurological disorder. See Table 1 for demographic information of the sample.

**Table 1:** Demographics and musical experience of sample (*N* = 255).

Demographic	Frequency	%
*Gender*		
Male	105	41.2
Female	144	56.5
Other	5	2.0
Not disclosed	1	0.4

*Education*		
Secondary school/GCSE/NVQ	17	6.7
A Level	8	3.1
Further/Adult Education	25	9.8
Undergraduate Degree	105	41.2
Master’s Degree	66	25.9
PhD/Doctoral Degree	32	12.5
Not disclosed	2	0.8

Hearing impairment*		
Hearing loss	30	11.8
Tinnitus	15	5.9
None	213	83.5

Psychiatric diagnosis*		
None	146	57.3
Depression	66	25.9
Schizophrenia/schizoaffective disorder	6	2.4
Anxiety	33	12.9
Bipolar disorder	12	4.7
ADHD	13	5.1
ASD	3	1.2
OCD	7	2.7

Neurological disorder*		
None	232	91.0
Epilepsy/seizures	5	2.0
Migraine	5	2.0
Other	12	4.7

*Musical experience*		
Non-musician	35	13.7
Music-loving non-musician	75	29.4
Amateur musician	74	29.0
Serious amateur musician	39	15.3
Semi-professional musician	23	9.0
Professional musician	9	3.5

* Participants could provide more than one response.

### 2.2 Measures

#### 2.2.1 Previous musical experience

Preliminary questions asked about musical expertise, musical preference, practising and listening habits. See Appendix 1 for a full list of questions.

#### 2.2.2 Phenomenology of Inner Music Questionnaire

The Phenomenology of Inner Music Questionnaire was designed as a preliminary exploration of the experience of MH, musical imagery, and earworms, including items based on previous literature on MH, but also items that have only been used in relation to imagery and earworms. Firstly, based on short definitions, participants were asked to classify their inner music as either musical imagery, earworm, or musical hallucination. A free text box was also provided for participants to expand on this description. Based on previous research into inner music or MH, further questions asked about aspects of the experience encompassing frequency, duration, level of detail (melody, harmony, intensity, presence of instruments, presence of lyrics), familiarity, effects on mood and behavior, amount of perceived control, triggers, and likelihood of being mistaken for an external stimulus. All questions required the participant to respond on a Likert scale, although many questions also provided a free text box to elicit more detailed responses. (See Appendix 1 for full questionnaire.)

#### 2.2.3 White Bear Suppression Inventory (WBSI)

The WBSI is a 15-item scale designed to measure the tendency to suppress unwanted thoughts. Various studies have implied different factor structures underlying the WBSI; here, we used the subscales identified and used by Muris, Merckelbach, & Horselenberg (1996), assessing tendency to have intrusive thoughts (e.g., *I have thoughts that I cannot stop*) and thought suppression (e.g. *I always try to put problems out of my mind*). For each question, participants are required to rate their agreement on a Likert scale from 1 (*Strongly Disagree*) to 5 (*Strongly Agree*). These subscales have previously been shown to have acceptable internal reliability (Jones & Fernyhough, 2006).

#### 2.2.4 Revised Launay-Slade Hallucination Scale (LSHS-R, auditory items)

Tendency to experience auditory hallucinations was assessed using the 5-item LSHS-R (McCarthy-Jones & Fernyhough, 2011; revised from Morrison et al., 2000) (e.g., *I hear people call my name and find that nobody has done so*). For each question, participants are required to indicate agreement with each question on a 4-point Likert scale, ranging from 1 (*Never*) to 4 (*Almost Always*). Total score can range from 5 to 20. It has previously shown high internal reliability (Cronbach’s α = .73) (McCarthy-Jones & Fernyhough, 2011).

#### 2.2.5 Varieties of Inner Speech Questionnaire (VISQ)

The VISQ is an 18-item scale designed to assess phenomenological features of inner speech. It consists of four subscales: evaluative inner speech (e.g., *I think in inner speech about what I have done, and whether it was right or not*), dialogic inner speech (e.g., *I talk back and forward to myself in my mind about things*), other people in inner speech (e.g., *I experience the voices of other people asking me questions in my head*) and condensed inner speech (e.g., *I think to myself in brief phrases and single words, rather than full sentences*). Each item is scored on a Likert scale from 1 (*Never*) to 6 (*All of the time*). Each subscale has previously shown high internal reliability (Cronbach’s α > .8) and acceptable test-retest reliability (>.6) (McCarthy-Jones & Fernyhough, 2011).

### 2.3 Data analysis

Given that our main area of interest regarded the phenomenological differences between MH and other forms of inner music (musical imagery, earworms), participants were categorized by the main type of experience they reported. If participants indicated in the free text box that they frequently experienced more than one form of inner music (for example, reporting both frequent MH and earworms), but indicated in the free text box that one of these was much more prevalent than the other, they were categorized according to their most prevalent experience. If participants indicated that they experienced more than one type of music, but did not report relative frequencies, they were categorized in a separate ‘mixed experiences’ group. This categorization was performed separately by two of the authors (PM, BA, k = .77), and any disagreements (*n* = 9) were resolved by discussion between authors. Thus, the sample was split into four groups (musical imagery, earworms, MH, mixed experiences) for between-group analysis.

We used chi-square analysis to investigate associations between type of musical experience and presence of a psychiatric/neurological diagnosis, hearing impairment, and level of musical expertise, and further explored significant results using odds ratios (OR) in conjunction with 95% confidence intervals. Due to mainly ordinal and non-normally distributed data, non-parametric ANOVAs (Kruskal-Wallis) were used to test for differences between the categories of inner music, for each phenomenological attribute. Where appropriate, Mann-Whitney *U* tests were used to investigate differences between MH and other individual categories; note that post-hoc tests were only conducted between MH and other categories (as opposed to between all different categories) to limit the number of tests performed. Based on our areas of interest, we split the analysis into four main sections: (1) demographic and etiological information; (2) basic characteristics and acoustic details of inner music; (3) location and controllability of inner music; (4) effect of inner music on behavior and mood. Qualitative examples given by participants are included throughout as illustrative examples of different aspects of their phenomenology. (All qualitative examples given are taken from participants in the MH group.) Bonferroni corrections for multiple comparisons were applied within each section (e.g., in Section 3.2, 10 tests are performed, so the alpha level is corrected to .05/10 = .005; in Section 3.3, 5 tests are performed so the alpha level is corrected to .05/5 = .01; in Section 3.4, 11 tests are performed, so the alpha level is corrected to .05/11 = .0045). Missed items in the VISQ, LSHS-R or WBSI were replaced with the mean from other items in the same (sub)scale.

## 3 Results

### 3.1 Demographics and categories of inner music

The first question of the Inner Music Questionnaire asked people to choose the category that best described the music they heard. Of the 255 participants, 17.3% reported musical hallucinations, 40.4% reported earworms, 27.1% reported musical imagery, whilst 15.3% were categorized in the ‘mixed experiences’ group. To investigate phenomenological differences between MH and other musical experiences, ‘inner music category’ was used as a between-subject variable. Only 1.2% of participants reported that they could ‘Never’ accurately describe their inner music (in response to Q5) but, since they continued to provide responses to the questions, these participants were not excluded from the sample. Table 1 summarizes basic demographics of the sample, whilst Table 2 shows these basic demographics broken down by the category of inner music reported by the participant. For the main effect of inner music type in this section, a Bonferroni-corrected alpha level of .05/9 = .006 was used.

**Table 2:** Demographics of sample by reported inner music category.

	MH	Imagery	Earworm	Mixed	*p*
*Basic demographics*					
N (% of sample)	17.3	27.1	40.4	15.3	
Gender (% female)	68.2	53.7	54.5	62.2	.369
Age	37.9 (13.5)	37.1 (13.1)	43.5 (13.3)	34.4 (10.5)	<.001*
Hearing impairment (%)	16.3	11.8	17.5	17.9	.755
Psychiatric diagnosis (%)	41.9	36.8	39.6	56.4	.228
Neurological diagnosis (%)	11.4	7.2	9.8	5.1	.714

*Musical expertise*					
Musicians (%)	31.8	63.8	54.4	79.5	<.001*
No. instruments played	1.5 (1.8)	2.4 (1.9)	2.0 (1.8)	3.3 (2.9)	<.001*
Hours practiced	2.2 (3.5)	4.5 (5.4)	2.04 (3.3)	4.0 (5.9)	.003*
Hours listened	9.5 (14.1)	9.4 (9.6)	8.9 (8.8)	9.5 (8.4)	.526

Musicians: % participants categorizing themselves as amateur, serious amateur, semi-professional, or professional musicians. No. instruments played: number of instruments participants reported playing (M, SD). Hours practiced: number of hours spent practicing music, per week (M, SD). Hours listened: number of hours participants reported actively listening to music per week.

* Significant at corrected alpha level (.05/9 = .0056).

A chi-square analysis indicated that there was not a significant association between presence of a psychiatric diagnosis (χ^2^(4) = 4.33, *p* = .228, *φ* = .131) or neurological diagnosis (χ^2^(3) = 1.36, *p* = .714, *φ* = .073) and the category of inner music. There was also no significant association between presence of a hearing impairment and category of inner music (χ^2^(3) = 0.89, *p* = .828, *φ* = .059).

There was a significant association between level of musical expertise and type of inner music reported (χ^2^(15) = 36.93, *p* = .001, *φ* = .381), although in this analysis 25% of cells had an expected count of < 5, due to the low number of semi-professional (*n* = 23) or professional (*n* = 9) musicians (at least compared to other groups) in the sample. This violates a key assumption of chi-square analysis (Howell, 2010) and, as such, the sample was collapsed into two groups: non-musicians (non-musicians and music-loving non-musicians; *n* = 110) and musicians (amateur, serious amateur, semi-professional, and professional musicians; *n* = 145). Again, a chi-square analysis indicated an association between musical expertise and inner music type (χ^2^(3) = 20.99, *p* < .001, *φ* = .287). Further analysis suggested that musicians were less likely to report MH (OR = 0.27, 95% CI [0.14–0.54]), but more likely to report mixed experiences (OR = 3.23, 95% CI [1.42, 7.33]) compared to non-musicians. There was little difference in the proportion of musicians in either the imagery (OR = 1.37, 95% CI [0.78, 2.41]) or earworm (OR = 0.78, 95% CI [0.47, 1.28]) groups. Non-parametric ANOVAs (Kruskal-Wallis) with inner music category as the independent variable suggested a similar pattern of results for number of hours spent practicing musical instruments per week (χ^2^(3) = 17.82, *p* < .001), with MH being associated with less music practice than imagery (U = 1084.5, *p* = .011), although not significantly different to earworms (U = 2168.5, *p* = .806) or mixed experiences (U = 623.5, *p* = .040) at the corrected alpha level. However, this pattern of results seemed to be specific to actually practising music, and did not hold for time spent listening to music (χ^2^(3) = 2.23, *p* = .526).

### 3.2 Basic characteristics of musical hallucinations

The questionnaire asked about a number of basic characteristics of inner music experiences, such as style of music (Q4), frequency (Q2), duration (Q3), familiarity (Q10), repetitiveness (Q22), and whether music was experienced in its entirety or was shortened (Q20 and 21). Participants were also asked whether their inner music included attributes such as melody, harmony, intensity, instruments, and lyrics (Q6). Table 3 presents descriptive statistics and the results of group contrasts, for the basic characteristics and acoustic details of the different categories of inner music. For main effects of inner music type, a Bonferroni-correct alpha level of .005 (.05/10) was used for all statistical tests in Section 3.2.

**Table 3:** Basic characteristics (*M, SD*) and acoustic details of inner music, by category.

	MH	Imagery	Earworms	Mixed	*p*	MH ≠ Imagery	MH ≠ Earworm	MH ≠ Mixed
*Basic characteristics (M, SD)*								
Frequency	2.86 (1.27)	1.62 (0.75)	1.81 (0.92)	1.87 (1.06)	<.001*	<.001^†^	<.001^†^	<.001^†^
Duration	1.66 (1.08)	1.49 (0.99)	1.92 (1.14)	1.92 (1.24)	.009	–	–	–
Familiarity	2.91 (1.41)	4.16 (0.74)	4.46 (0.62)	3.67 (0.87)	<.001*	<.001^†^	<.001^†^	.007^†^
Shortened	3.21 (1.23)	3.36 (1.15)	3.64 (1.03)	3.53 (0.73)	.151	–	–	–
Repetitive	2.82 (1.24)	3.33 (0.68)	3.63 (0.84)	3.37 (0.82)	<.001*	.011^†^	<.001^†^	.032
Complete tune	2.86 (1.25)	2.58 (1.06)	2.34 (1.13)	2.49 (0.96)	.123	–	–	–

*Acoustic detail (%)*								
Melody	97.1	99	95.5	94.9	–	–	–	–
Harmony	81.2	73.8	72.7	84.6	.389	–	–	–
Intensity (loud/soft)	73.9	60.2	61.4	74.4	.163	–	–	–
Instrument	79.7	75.7	65.9	87.2	.128	–	–	–
Lyrics	79.7	83.5	47.7	79.5	< .001*	–	–	–

Basic characteristics: Frequency (Q2), Duration (Q3). Familiarity (Q10), Shortened (Q21), Repetitive (Q22), Complete tune (Q20), Acoustic details (Q6). The *p* values provide the result of non-parametric ANOVAs (Kruskal-Wallis) for ordinal data, or chi square analysis for categorical data. The final three columns indicate significance level for contrasts between MH and the other groups (Mann-Whitney). Note that larger numbers for ‘Frequency’ denote less frequent experiences.

* Significant at corrected alpha level (.05 / 10 = .005). ^†^ Between group contrast significant at corrected alpha level (.05 / 3 = .017).

Classical music was the most frequently reported style of inner music in participants with MH (47.7%), whereas rock music was the most frequently reported style in the musical imagery group (64.2%), and pop music in the earworms group (66.0%). The mixed experiences group reported that both pop music and classical music were the most frequent style of their inner music (both 62.5%). See Table 4 for a full list of reported music styles and their frequency.

**Table 4:** Reported genres of inner music, by inner music category (%).

Music style	MH	Imagery	Earworm	Multiple
Dance	18.2	23.9	25.2	45.0
Contemporary	22.7	32.8	41.7	42.5
Pop	34.1	61.2	66.0*	62.5*
Chart	13.6	16.4	25.2	32.5
Classical	47.7*	58.2	52.4	62.5*
Opera/choral	15.9	40.3	40.8	52.5
Soul	6.8	22.4	21.4	40.0
Orchestral	22.7	35.8	32.0	55.0
Rock	38.6	64.2*	56.3	52.5
Instrument/chamber	20.5	35.8	33.0	52.5
Folk	25.0	40.3	40.8	47.5
Jazz	18.2	26.9	27.2	50.0
Other	47.7	37.3	37.9	60.0

Participants were asked to indicate the musical style/genre that their inner music took. They could choose as many genres as they wanted. Numbers represent percentage of participants in each group that chose each genre.

* Most frequently reported genre per inner music category.

A non-parametric (Kruskal-Wallis) ANOVA with inner music frequency (Likert scale responses; see Appendix 1) as the dependent variable showed a significant main effect of inner music category (χ^2^(3) = 33.73, *p* < .001), with Mann-Whitney *U* tests (Bonferroni corrected alpha levels at .05/3 = .017) indicating that participants in the MH group reported the experience as occurring significantly less frequently than those in the imagery group (*U* = 651.5, *p* < .001), earworm group (*U* = 1153, *p* < .001), or the mixed experiences group (*U* = 468, *p* < .001). There was also a significant main effect of inner music type on familiarity (χ^2^(3) = 56.96, *p* < .001), with MH being rated as less familiar than imagery (*U* = 738, *p* < .001), earworms (*U* = 866, *p* < .001), or mixed experiences (*U* = 573.5, *p* < .001). For example, a typical response from a participant in the MH group in a free-text box was:

“*I can tell the style, but it’s not songs I’ve heard before. It’s new songs*.”

There was also a main effect on repetitiveness (χ^2^(3) = 21.22, *p* < .001), with MH being reported as less repetitive than earworms (*U* = 1341, *p* < .001) or imagery (*U* = 1117, *p* = .011), but not significantly different to the mixed experiences group (U = 618, *p* = .032), following corrections to the alpha level (.05/3 = .017). There was also no significant effect of inner music type on duration (χ^2^(3) = 11.60, *p* = .009), or the extent to which participants reported inner music being shortened (χ^2^(3) = 5.31, *p* = .151) or a complete song (χ^2^(3) = 5.77, *p* = .123).

Almost all participants reported being able to perceive melody in inner music (97.3% in overall sample), with most also being able to perceive harmony (77.4%), intensity (66.5%), instruments (77.0%) and lyrics (75.9%). Chi-square analysis indicated no association between inner music category and whether harmony (χ^2^(3) = 3.02, *p* = .389, *φ* = .109), intensity (χ^2^(3) = 5.12, *p* = .163, *φ* = .142) or instruments (χ^2^(3) = 5.69, *p* = .128, *φ* = .149) were perceived, although there was an association between inner music type and whether lyrics were perceived (χ^2^(3) = 23.02, *p* = < .001, *φ* = .300). (Given that >97% of participants reported being able to perceive a melody, there was insufficient variation to test the association between inner music type and melody.) Further analysis showed that MH were less likely to include lyrics than other types of inner music (OR = 0.21, 95% CI [0.10, 0.41]), whereas earworms were more likely to include lyrics (OR = 2.13, 95% CI [1.14, 3.98]). Meanwhile, the imagery (OR = 1.37, 95% CI [0.70, 2.68]) and mixed experiences (OR = 1.29, 95% CI [0.56, 2.98] groups were no more or less likely to include lyrics. One participant who experienced MH commented:

*“...[they] tend not to involve voices but do involve many different instruments. I have occasionally heard other genres of music, including with voices, but I have not been able to understand the lyrics.”*

### 3.3 Perceived control, location, and anticipation of musical hallucinations

Further questions asked whether participants felt control over their inner music (Q23), whether they could anticipate the experience (Q24), whether they knew the trigger of the experience (Q25), whether they ever mistook it for an externally located stimuli (Q26), and whether they felt like the experience was of their own creation (Q11). Table 5 summarizes responses from these questions by category of inner music. For main effects of inner music type, a Bonferroni-corrected alpha level of .01 (.05/5) was used for all statistical tests in Section 3.3.

**Table 5:** location and controllability (*M, SD*).

	MH	Imagery	Earworm	Mixed	*p*	MH ≠ Imagery	MH ≠ Earworm	MH ≠ Mixed
Uncontrollability	2.57 (1.49)	3.35 (0.95)	2.91 (1.03)	3.00 (0.95)	.009*	.003^†^	.129	.137
Anticipation	1.95 (1.27)	1.73 (1.08)	1.86 (1.29)	1.92 (1.12)	.727	–	–	–
Mistaken for external	2.70 (1.25)	1.25 (0.55)	1.22 (0.50)	2.00 (0.73)	<.001*	<.001^†^	<.001^†^	.008^†^
Own creation	2.86 (1.25)	2.42 (0.90)	1.93 (0.84)	2.90 (0.75)	<.001*	.030	<.001^†^	.973
Knowledge of trigger (%)	50.0	35.8	40.8	36.8	.481	–	–	–

Uncontrollability (Q23), Anticipation (Q24), Mistaken for external (Q26), Own creation (Q11), Knowledge of trigger (Q25). The *p* values provide the result of non-parametric ANOVAs (Kruskal-Wallis) for ordinal data, or chi square analysis for categorical data. The final three columns indicate significance level for contrasts between MH and the other groups (Mann-Whitney).

* Significant at corrected alpha level (.05/5 = .01). ^†^Between group contrast significant at corrected alpha level (.05/3 = .017).

There was a main effect of category of inner music on perceived control (χ^2^(3) = 11.52, *p* = .009), with participants reporting MH also reporting less control over the experience compared to participants in the imagery group (*U* = 1033, *p* = .003). However, there was no significant difference between perceived control of MH and earworms, or between MH and mixed experiences (*p*s > .129). A typical comment related to the perceived effortlessness of MH, with little or no control:

“*Imagination to me is a conscious effort – this isn’t*.”

“*Like a radio station, I just have to wait for the next song*.”

Others, meanwhile, described techniques to stop the MH, such as distracting themselves with another activity:

“*I just think of something specific as a distraction (e.g., I’m thinking about typing this response correctly, so my internal background music is switched off for now*).”

When asked about the frequency with which their inner music was mistaken for coming from the external environment, there was a significant main effect of inner music category (χ^2^(3) = 95.10, *p* < .001), with MH being more likely to be mistaken for an external percept than imagery (*U* = 460, *p* < .001), earworms (*U* = 656, *p* < .001) and mixed experiences (*U* = 581, *p* = .008). For example:

*“There is usually a moment where I am not entirely sure if it is external or internal, but the quality of sound and the apparent feeling of “proximity” is strange when it is a hallucination. In other words, it might sound soft as if it should be coming from far away, and yet it does not sound as though it is coming through any barriers like walls... it is almost as if it is coming through earphones, closer to me than the outside world, but not exactly ‘in my head’”*

“*Remembering music is like a faint shadow of real music...whereas hallucinating it really involves hearing it*”

There was a significant main effect of inner music type on the extent to which participants reported that their inner music was their own creation (χ^2^(3) = 39.09, *p* < .001), with participants in the MH group, counterintuitively, rating this attribute more highly than participants in the earworm group (*U* = 1255, *p* < .001), although there was no difference between the MH and imagery or mixed experiences groups at the corrected alpha level (*p*s > .30).

There was no main effect of inner music category for reports of being able to anticipate the experience or knowing what triggered the experience (see Table 5 for statistics).

### 3.4 Effects of musical hallucinations on mood and behavior

Participants were asked a number of questions about the effect of inner music on their behavior and mood, including whether they were able to hum along with the inner music (Q8), whether their body moved with the music they experienced (Q9), whether the experience reflected their own feelings (Q18), or was associated with negative affect (Q15) (see Table 6). A Bonferroni-corrected alpha level of .05/11 = .0045 was used for all statistical tests in Section 3.4.

**Table 6:** effects of inner music on behavior and mood.

	MH	Imagery	Earworm	Mixed	*p*	MH ≠ Imagery	MH ≠ Earworm	MH ≠ **Mixed**
*Effects on behaviour (M, SD)*								
Can hum along	2.95 (1.51)	4.18 (0.99)	4.15 (0.97)	4.08 (0.87)	<.001*	<.001^†^	<.001^†^	.001^†^
Can hum after	2.91 (1.51)	4.03 (1.11)	4.03 (0.98)	3.72 (0.97)	<.001*	<.001^†^	<.001^†^	.012^†^
Body moves to music	2.02 (1.14)	3.03 (0.98)	2.73 (1.01)	3.11 (1.01)	<.001*	<.001^†^	<.001^†^	<.001^†^
Affects relationships with others	1.40 (0.70)	1.49 (0.67)	1.44 (0.75)	1.47 (0.61)	.580	–	–	–

*Effects on mood (M, SD)*								
Inner music changes mood	2.42 (1.05)	2.82 (0.95)	2.72 (0.85)	2.78 (0.76)	.238	–	–	–
Makes me feel anxious	1.77 (1.02)	1.56 (0.76)	1.98 (1.02)	1.86 (0.72)	.079	–	–	–
Makes me feel excited	2.19 (1.14)	2.76 (0.92)	2.24 (0.92)	2.44 (0.74)	.002*	.019	.929	.253
Makes me feel depressed	1.44 (0.80)	1.69 (0.82)	1.81 (0.87)	1.81 (0.82)	.042	–	–	–
Inner music is pleasant	3.58 (1.30)	3.82 (0.71)	3.58 (0.73)	3.69 (0.86)	.094	–	–	–
Calms me down	2.21 (1.21)	2.48 (1.08)	2.18 (1.09)	2.53 (1.00)	.078	–	–	–
Reflects my feelings	2.02 (1.21)	2.92 (1.06)	2.61 (1.01)	2.92 (1.03)	<.001*	<.001^†^	.003^†^	.002^†^

Effects on behavior: Can hum along (Q7), Can hum after (Q8), Body moves to music (Q9), Affects relationships with others (Q19). Effects on mood: Inner music changes mood (Q12), Makes me feel anxious (Q13), Makes me feel excited (Q14), Makes me feel depressed (Q15), Inner music is pleasant (Q16), Calms me down (Q17), Reflects my feelings (Q18).

* Significant at corrected alpha level (.05/11 = .0045). ^†^Between group contrast significant at corrected alpha level (.05/3 = .017).

The extent to which participants reported that the inner music reflected how they were feeling differed between categories (χ^2^(3) = 19.22, *p* < .001), with MH being rated lower on this attribute than imagery (*U* = 825.5, *p* < .001), earworms (*U* = 1539, *p* = .003) or the mixed experiences group (*U* = 522, *p* = .002). Although there was a significant main effect of inner music category on the extent to which the experience made the participant feel excited (χ^2^(3) = 15.30, *p* = .002), further tests did not reveal any significant differences at the corrected alpha level between MH and any other categories (all *p*s > .019). Notably, there was no significant effect of inner music category on the experience contributing to the participant feeling depressed (χ^2^(3) = 8.21, *p* = .042) or anxious (χ^2^(3) = 6.79, *p* = .079) after corrections to the alpha level, providing no strong evidence that MH were associated with negative affect more than other forms of inner music. For example:

“*It’s never anything emotional playing, usually just ‘there’. I’ve gotten used to it for the most part, but the most it makes me feel is annoyed*.”

Participants reported being less able to hum along with MH (χ^2^(3) = 24.72, *p* < .001), compared to imagery (*U* = 777.5, *p* < .001), earworms (*U* = 1204.5, *p* < .001), or mixed experiences (*U* = 481, *p* = .001), as well as being less able to hum the music after the experience (χ^2^(3) = 22.15, *p* < .001) compared to imagery (*U* = 860.5, *p* < .001), earworms (*U* = 1313, *p* < .001), or mixed experiences (*U* = 589, *p* = .012). Participants also reported moving their body less to MH (χ^2^(3) = 28.88, *p* < .001), compared to imagery (*U* = 736, *p* < .001), earworms (*U* = 1442, *p* < .001), or mixed experiences (*U* = 416, *p* < .001). One typical comment was:

“*Unfamiliar, no lyrics, no compulsion to sing or hum along*”

Finally, there was no association between inner music category and the extent to which it was said to affect the participant’s relationship with others (see Table 3 for statistics).

### 3.5 Associations between musical hallucinations, inner speech phenomenology, and intrusive thoughts

Participants also completed a small number of quantitative self-report measures relating to phenomenology of inner speech (VISQ), intrusive thoughts and thought suppression (WBSI), and auditory hallucination-proneness (LSHS). There was no main effect of inner music type on any measure of inner speech phenomenology, intrusive thoughts, or thought suppression (all *p*s > .092). Unsurprisingly, there was a main effect of inner music type on hallucination-proneness (χ^2^(3) = 20.03, *p* < .001), with participants in the MH category (*M* = 9.60, *SD* = 2.61) scoring higher than those in the imagery category (*M* = 8.18, *SD* = 2.50, *U* = 958, *p* = .005) and the earworm category (*M* = 8.19, *SD* = 2.31, *U* = 1445.5, *p* = .003), although not significantly differently to the mixed experiences category (*M* = 10.21, *SD* = 3.22, *U* = 723.5, *p* = .469).

## 4 Discussion

The present study provided preliminary evidence for a number of phenomenological differences between MH and other forms of inner music (summarized in Table 7). Whilst some of these differences fit closely with the typical conceptualization of hallucinatory experience (e.g., experienced as externally located, uncontrollable), others were less expected and may open avenues for future research. For example, the data suggested that MH are less likely to be experienced by musicians, less repetitive, less likely to include lyrical content, and are described as less likely to be associated with one’s own feelings, compared to musical imagery or earworms. Somewhat counterintuitively, the data also suggested that MH were more likely to be experienced as one’s ‘own creation’ compared to earworms. Importantly, our findings suggest that there are key differences between, in particular, MH and earworms. Moreover, given the self-report methodology used in this study, the data provides valuable insight into how people make judgements about their own experiences (that is, what do people classify as hallucinatory?). Here, the findings are compared to previous literature on MH, but several new findings that could form the basis for future research are also interpreted.

**Table 7:** Summary of findings.

Musical hallucinations, compared to musical imagery and earworms, are:
*Less*
Often experienced by musicians
Frequent
Familiar
Repetitive
Likely to include lyrics
Reflective of one’s own feelings
Easy to hum along with, or hum afterwards
Likely to be accompanied by body movements
Controllable*

*More*
Likely to be mistaken for an external stimulus
Likely to be experienced as one’s 'own creation'**

* Difference only between MH and imagery.

** Difference only between MH and earworms.

A previous systematic review by Cope and Baguley (2009), focusing on etiological factors underlying MH, indicated that hearing loss, female gender, old age, and social isolation were all risk factors for MH. The present data did not indicate that females were more likely to report MH; while the proportion of females in the MH category was higher than other groups, this was not statistically significant. The data did not show elevated levels of hearing impairment in individuals reporting MH as opposed to other forms of inner musical experience, although, given the relatively low rates of reported impairment in our sample, this may be an issue of statistical power. Numerous studies have previously linked AH or MH to hearing impairment (Cope and Baguley, 2009, Griffiths, 2000, Kumar et al., 2014, Linszen et al., 2018); one interesting question, therefore, would be to examine the phenomenology of MH that seem to be linked to hearing loss, compared to those that are not. Rates of psychiatric or neurological diagnoses were also similar across different forms of reported inner music, and, furthermore, individuals with MH were not significantly more likely to say that the experience made them depressed or anxious, compared to individuals reporting other forms of inner music. Previous literature has provided mixed evidence regarding negative emotions associated with MH. The most prevalent emotional description in Saba and Keshavan (1997) study was ‘soothing’; in contrast, Evers and Ellger (2004) found that 41% of participants described the experience as ‘frightening’. Such differences may partially reflect the different populations from which data was collected, with most previous research investigating MH in psychiatric or neurological patients. Our data, meanwhile, is consistent with the view that MH, and auditory hallucinations more broadly, can occur without significant distress and outside of any need for care (Johns et al., 2014).

Indeed, the only demographic factor significantly associated with MH in the present data was level of musical expertise, with individuals reporting MH being less likely to classify themselves as a musician, playing fewer musical instruments, and spending less time practising music. Previous research has suggested that musical training is associated with superior performance on tasks requiring auditory imagery generation, for example to complete a musical sequence (Aleman, Nieuwenstein, Böcker, & de Haan, 2000), or to evoke spontaneous experiences of musical imagery, and importantly, voluntarily modify the experiences (Goycoolea et al., 2007). Similarly, Pallesen et al. (2010) showed that individuals with musical training showed increased performance on an auditory working memory task, showing greater levels of cortical activation in areas of the brain typically associated with cognitive control, such as the lateral prefrontal cortex and anterior cingulate cortex. It is possible, therefore, that enhanced cognitive control among musicians may contribute to the decreased prevalence of MH, although further research is needed to investigate this issue.

One of the few studies to report phenomenological details of MH (Saba & Keshavan, 1997) reports data from 100 participants with a diagnosis of schizophrenia, finding that 16 of these individuals experienced MH. These experiences were not compared directly to experiences of musical imagery or earworms; consistent with our findings, however, a substantial number were rated as being perceived as emanating from the external environment, and approximately half were rated as not under volitional control of the individual. Saba and Keshavan, however, argued that if the individual reported any volitional control of the experience, it should not be defined as an MH, suggesting that this lack of control is actually a key feature of the experience. This is in contrast to our data, which suggests that while MH were rated as much lower in controllability than musical imagery, earworms were rated intermediately between the two categories. One possibility consistent with Saba and Keshavan’s argument is that the experience of volitional control can vary along a continuum, with imagery becoming hallucinatory when it is extremely uncontrollable. Level of volitional control may also be an important factor in clinical distress, with recent studies showing that individuals that report regular AVH but have no clinical diagnosis report higher levels of control, compared to those with a clinical diagnosis (Alderson-Day et al., 2017, Daalman et al., 2011, Powers et al., 2016). On this view, earworms would simply fall on a midpoint of this continuum between musical imagery and MH. However, our findings also suggest that MH differ in a number of other ways from both musical imagery and earworms; for example, MH are much more likely to be experienced as externally located, consistent with the argument made by Williams (2015). Indeed, given that a substantial proportion of individuals in the Saba and Keshavan study (37.5%) perceived their MH as externally located, it is unclear why only volitional control, rather than perceived location, was chosen as the main criteria by which MH were defined.

Another clear difference between earworms and MH, in our sample, was the reported familiarity of the perceived music, with MH being rated as less familiar to the participant than other forms of inner music. It is possible that this feeling of unfamiliarity may add to a feeling of alienness typically associated with hallucinations, as opposed to musical imagery or earworms. In contrast, previous studies have reported that as many as 78% of cases of MH were experienced as familiar music (Evers & Ellger, 2004). There are two possible methodological differences that may account for this discrepancy. Firstly, as already mentioned, the two referenced studies focused mainly on MH occurring in psychiatric and neurological disorders, whereas rates of diagnoses were much lower in the current sample. As such, our data may reflect MH occurring across a broader population, mainly consisting of those without a need for care, and without comorbid psychiatric symptoms or brain damage. Secondly, previous studies have deliberately excluded participants reporting ‘pseudohallucinations’ (which Evers and Ellger dismiss as being linked to ‘memory representations’), although this distinction is no longer typically used in hallucinations research. As such, the present data presumably encompasses a wider variety of experiences regarded as hallucinatory; indeed, given the self-report nature of this data, a sufficient level of insight regarding the hallucinatory nature of their experience was necessary for participants to report on the features of their MH.

Our data suggested that less than half of participants reported the presence of lyrics in their MH, compared to rates of approximately 80% in other forms of inner music. This is, again, in contrast to Saba and Keshavan, who noted that MH consisting only of instrumental music was rare. It should be noted that the present questionnaire only asked about lyrical content rather than concurrent MH and AVH. Nevertheless, the low frequency of lyrical content was an unexpected finding, and highlights a key difference between MH and AVH, the most frequent form of auditory hallucination. Given that cognitive neuroscientific models of AVH specify a key role for brain networks involved in speech production and perception (Allen et al., 2008, Moseley et al., 2013), this is suggestive that non-lyrical MH may be associated with different cognitive mechanisms than AVH, rather than simply differing in content. An interesting area for future research would be to investigate differences between the phenomenology and cognitive mechanisms underlying MH with and without lyrics, as well as investigating the prevalence and phenomenology of mixed AVH and MH.

A further unexpected, and rather counterintuitive, aspect in which MH differed from other forms of inner music was in rating the extent to which the music was one’s own creation. Although MH were rated as lower in controllability and familiarity, they were actually rated as significantly higher on this attribute, in comparison to earworms. A frequent argument is that earworms occur due to unintentional re-activation of memory representations of previously heard music (Kvavilashvili and Mandler, 2004, Liikkanen, 2012). In this sense, they may be viewed as not one’s own creation, since the individual realizes that they are elicited by an external stimulus (that is, the individual is aware that they were not the author of the music). In contrast, a less clear link with previously heard music may lead to recognition of creative ownership of MH, despite a lack of controllability or familiarity of the music. This argument assumes a fairly high level of insight regarding one’s MH; that said, participants in this sample presumably required sufficient levels of insight to self-classify their experiences as hallucinatory. It is particularly interesting that volitional control and authorship can be dissociated in this way, which potentially provides evidence for a dissociation between individual sense of agency and sense of ownership in relation to MH (Gallagher, 2000); that is, our data suggest that individuals may feel a lower sense of agency, but a retained sense of ownership, over MH.

This study, then, has provided data on several aspects of the phenomenology of MH. In comparison to the most widely studied type of inner music – earworms – MH are less controllable, more likely to be experienced as coming from the external environment, less familiar in content, less repetitive, less likely to include lyrics, and more recognizable as one’s own creation. These differences highlight a need for greater clarity in the use of definitions when talking about different experiences of inner music. Our data is not consistent with previous claims that MH can simply be thought of as earworms that have reached pathological levels (e.g., Hemming, cited in Williams, 2015), for two reasons: firstly, MH appear to differ from earworms on a number of phenomenological attributes, rather than simply being more persistent or distressing; secondly, many individuals in our sample reported experiencing MH without having a hearing impairment, psychiatric or neurological disorder, or any resulting distress. As such, more nuanced definitions of MH and earworms are needed. Previous research tends to have studied all involuntary musical imagery as one type of experience (Floridou et al., 2015, Müllensiefen et al., 2014, Williamson et al., 2014), with some conflating the terms INMI, earworms, and MH. Williams (2015) argues that the key difference between MH and earworms is the perception of spatial location, and suggests that INMI should be used as an umbrella term which can be further separated into earworms and MH. Our data support the importance of spatial location, but also suggest that this is not the sole difference, with aspects such as repetitiveness and familiarity also appearing to be important. Other instances of musical experience not included in this survey should also be investigated, including musical pareidolia (hearing music in other sounds) and musical memories.

Future research should further investigate some of the findings presented here. One limitation of the present study was that it required participants to classify their own experience as hallucinatory. Ideally, such experiences should be enquired about via a face-to-face clinical interview; however, online surveys can reveal experientially rich and sometimes unexpected aspects of hallucinatory phenomenology (see, for example, Woods et al., 2014, Woods et al., 2015). Additionally, although this study showed a link between a lack of musical training and MH, it is impossible to establish cause and effect from our data. Although findings of improved cognitive control following musical training are suggestive that this may reduce the likelihood of MH, a randomized controlled trial would be needed to make a direct link. Equally, it is possible that individuals who experience MH are less likely to pursue musical training, if their inner musical experiences are persistent or appraised negatively. Another important line of research will be to investigate the cognitive and neural correlates of MH, in comparison to musical imagery or earworms, about which little is currently known. An interesting question is whether (at least some) MH can be explained within a similar framework to AVH; that is, if MH can be explained as musical imagery that has been misattributed to an external source (Fernyhough, 2016). Studies investigating the association between MH and performance on tasks requiring the monitoring of self-generated actions could be the first step in this direction. Given the numerous phenomenological differences between musical imagery and MH highlighted above, however, a self-monitoring model may be too simplistic. Although research into MH is in its infancy, this study has provided preliminary evidence regarding a number of phenomenological aspects of MH that have not previously been addressed; future research should aim to replicate and extend these findings, which can be informative of inner musical experience in its many different forms.

## Declarations of interest

None.

## Footnotes

1 In this paper, the term ‘inner music’ will be used as an umbrella term, to refer to all forms of self-generated, internal musical experience (e.g., musical imagery, earworms, musical hallucinations).

## References

[b0005] Alderson-Day B., Lima C.F., Evans S., Krishnan S., Shanmugalingam P., Fernyhough C. (2017). Distinct processing of ambiguous speech in people with non-clinical auditory verbal hallucinations. Brain.

[b0010] Aleman A., Nieuwenstein M.R., Böcker K.B.E., de Haan E.H.F. (2000). Music training and mental imagery ability. Neuropsychologia.

[b0015] Allen P., Larøi F., McGuire P.K., Aleman A. (2008). The hallucinating brain: A review of structural and functional neuroimaging studies of hallucinations. Neuroscience & Biobehavioral Reviews.

[b0020] Bailes F. (2006). The use of experience sampling methods to monitor musical imagery in everyday life. Musicae Scientiae.

[b0025] Bailes F. (2007). The prevalence and nature of imagined music in the everyday lives of music students. Psychology of Music.

[b0030] Beaman C.P., Williams T.I. (2010). Earworms (stuck song syndrome): Towards a natural history of intrusive thoughts. British Journal of Psychology.

[b0035] Beaty R.E., Burgin C.J., Nusbaum E.C., Kwapil T.R., Hodges D.A., Silvia P.J. (2013). Music to the inner ears: Exploring individual differences in musical imagery. Consciousness and Cognition.

[b0040] Brodsky W., Henik A., Rubinstein B.-S., Zorman M. (2003). Auditory imagery from musical notation in expert musicians. Perception & Psychophysics.

[b0045] Cope T.E., Baguley D.m. (2009). Is musical hallucination an otological phenomenon? a review of the literature. Clinical Otolaryngology.

[b0050] Copolov D., Trauer T., Mackinnon A. (2004). On the non-significance of internal versus external auditory hallucinations. Schizophrenia Research.

[b0055] Daalman K., Boks M.P.M., Diederen K.M.J., de Weijer A.D., Blom J.D., Kahn R.S., Sommer I.E.C. (2011). The same or different?: A phenomenological comparison of auditory verbal hallucinations in healthy and psychotic individuals. The Journal of Clinical Psychiatry.

[b0060] Evers S., Ellger T. (2004). The clinical spectrum of musical hallucinations. Journal of the Neurological Sciences.

[b0065] Farrugia N., Jakubowski K., Cusack R., Stewart L. (2015). Tunes stuck in your brain: The frequency and affective evaluation of involuntary musical imagery correlate with cortical structure. Consciousness and Cognition.

[b0070] Fernyhough C. (2016). The Voices Within.

[b0075] Floridou G.A., Williamson V.J., Stewart L., Müllensiefen D. (2015). The Involuntary Musical Imagery Scale (IMIS). Psychomusicology: Music, Mind, and Brain.

[b0080] Gallagher S. (2000). Philosophical conceptions of the self: Implications for cognitive science. Trends in Cognitive Science.

[b0085] Golden E.C., Josephs K.A. (2015). Minds on replay: Musical hallucinations and their relationship to neurological disease. Brain.

[b0090] Goycoolea M.V., Mena I., Neubauer S.G., Levy R.G., Grez M.F., Berger C.G. (2007). Musical brains: A study of spontaneous and evoked musical sensations without external auditory stimuli. Acta Oto-Laryngologica.

[b0095] Griffiths T.D. (2000). Musical hallucinosis in acquired deafness. Brain.

[b0100] Hermesh H., Konas S., Shiloh R., Dar R., Marom S., Weizman A., Gross-Isseroff R. (2004). Musical hallucinations: Prevalence in psychotic and nonpsychotic outpatients. The Journal of Clinical Psychiatry.

[b0105] Howell D.C. (2010). Statistical Methods for Psychology.

[b0110] Johns L.C., Kompus K., Connell M., Humpston C., Lincoln T.M., Longden E., Larøi F. (2014). Auditory verbal hallucinations in persons with and without a need for care. Schizophrenia Bulletin.

[b0115] Jones S.R., Fernyhough C. (2006). The roles of thought suppression and metacognitive beliefs in proneness to auditory verbal hallucinations in a non-clinical sample. Personality and Individual Differences.

[b0120] Kumar S., Sedley W., Barnes G., Teki S., Griffiths T.D. (2014). A brain basis for musical hallucinations. Cortex.

[b0125] Kvavilashvili L., Mandler G. (2004). Out of one’s mind: A study of involuntary semantic memories. Cognitive Psychology.

[b0130] Liikkanen L.A. (2012). Musical activities predispose to involuntary musical imagery. Psychology of Music.

[b0135] Linszen, M. M. J., van Zanten, G. A., Teunisse, R. J., Brouwer, R. M., Scheltens, P., & Sommer, I. E. (2018). Psychological Medicine, 1–8. 10.1017/S0033291718000594. 29554989

[b0140] McCarthy-Jones S.R., Fernyhough C. (2011). The varieties of inner speech: Links between quality of inner speech and psychopathological variables in a sample of young adults. Consciousness and Cognition.

[b0145] McCarthy-Jones S., Trauer T., Mackinnon A., Sims E., Thomas N., Copolov D.L. (2012). A new phenomenological survey of auditory hallucinations: Evidence for subtypes and implications for theory and practice. Schizophrenia Bulletin.

[b0150] Morrison A.P., Wells A., Nothard S. (2000). Cognitive factors in predisposition to auditory and visual hallucinations. British Journal of Clinical Psychology.

[b0155] Moseley P., Fernyhough C., Ellison A. (2013). Auditory verbal hallucinations as atypical inner speech monitoring, and the potential of neurostimulation as a treatment option. Neuroscience & Biobehavioral Reviews.

[b0160] Müllensiefen D., Fry J., Jones R., Jilka S., Stewart L., Williamson V.J. (2014). Individual differences predict patterns in spontaneous involuntary musical imagery. Music Perception: An Interdisciplinary Journal.

[b0165] Muris P., Merckelbach H., Horselenberg R. (1996). Individual differences in thought suppression. The White Bear Suppression Inventory: Factor structure, reliability, validity and correlates. Behaviour Research and Therapy.

[b0170] Nayani T.H., David A.S. (1996). The auditory hallucination: A phenomenological survey. Psychological Medicine.

[b0175] Pallesen K.J., Brattico E., Bailey C.J., Korvenoja A., Koivisto J., Gjedde A., Carlson S. (2010). Cognitive control in auditory working memory is enhanced in musicians. PLOS ONE.

[b0180] Powers A., Kelley M., Corlett P. (2016). Varieties of voice-hearing: Psychics and the psychosis continuum. Schizophrenia Bulletin.

[b0185] Rocha S.C.M., Kii M.A., Pereira C.B., Borelli D.T., Forlenza O., Sanchez T.G. (2015). Multidisciplinary assessment of patients with musical hallucinations, tinnitus and hearing loss. Psychopathology.

[b0190] Saba P.R., Keshavan M.S. (1997). Musical hallucinations and musical imagery: Prevalence and phenomenology in schizophrenic inpatients. Psychopathology.

[b0195] Teunisse R.J., Olde-Rikkert M.G.M. (2012). Prevalence of musical hallucinations in patients referred for audiometric testing. The American Journal of Geriatric Psychiatry.

[b0200] Warner N., Aziz V. (2005). Hymns and arias: Musical hallucinations in older people in Wales. International Journal of Geriatric Psychiatry.

[b0205] Williams T.I. (2015). The classification of involuntary musical imagery: The case for earworms. Psychomusicology: Music, Mind, and Brain.

[b0210] Williamson V.J., Jilka S.R., Fry J., Finkel S., Müllensiefen D., Stewart L. (2012). How do “earworms” start? Classifying the everyday circumstances of Involuntary Musical Imagery. Psychology of Music.

[b0215] Williamson V.J., Liikkanen L.A., Jakubowski K., Stewart L. (2014). Sticky tunes: How do people react to involuntary musical imagery?. PLoS One.

[b0220] Woods A., Jones N., Alderson-Day B., Callard F., Fernyhough C. (2015). Experiences of hearing voices: Analysis of a novel phenomenological survey. The Lancet Psychiatry.

[b0225] Woods A., Jones N., Bernini M., Callard F., Alderson-Day B., Badcock J. (2014). Interdisciplinary approaches to the phenomenology of auditory verbal hallucinations. Schizophrenia Bulletin.

