# Supplementary material for: Musical hallucinations, musical imagery, and earworms: A new phenomenological survey
Source: Conscious Cogn. 2018 Oct;65:83–94. doi: 10.1016/j.concog.2018.07.009 (PMC6204882; doi:10.1016/j.concog.2018.07.009)
Supplement: Supplementary file 1 [file mmc1.docx]

| **Supplementary Materials: Phenomenology of Inner Music Questionnaire** | |
| --- | --- |
|  | **This questionnaire aims to explore experiences of “inner music”. This might include imagining or hearing music in your head (sometimes called musical imagery), having a tune stuck in your head (an “earworm”) or hearing music when no music is actually playing in the surrounding environment (a musical hallucination).**  **We are going to refer to these kinds of inner music as the music you hear in your head. Please use the scale to indicate whether the following statements apply to you or not, and how often they occur.**  * Question includes free text box for further detail  ** Question consists of free text response only  All questions included the following response options, unless otherwise specified: 1) Never; 2) Very occasionally; 3) Some of the time; 4) Most of the time; 5) All of the time. |
| 1 | How would you describe the music you hear in your head? 1) Musical imagery; 2) Earworm; 3) Musical hallucination; 4) Other (please specify)* |
| 2 | On average I hear music in my head, 1) At least once per hour; 2) At least once per day; 3) At least once per week; 4) At least once per month; 5) Less often than once per month |
| 3 | On average the music I hear in my head lasts for, 1) Minutes; 2) Hours; 3) Days; 4) Weeks; 5) Months/longer |
| 4 | How would you describe the style of music you hear in your head (please tick all that are appropriate), 1) Dance; 2) Contemporary; 3) Pop; 4) Chart; 5) Classical; 6) Opera/choral/vocal; 6) Soul; 7) Orchestral; 8) Rock; 9) Solo instrument/chamber; 10) Folk; 11) Jazz; 12) Other |
| 5 | I can accurately describe my music-hearing experiences* |
| 6 | In how much detail can you hear the music in your head? Can you hear: 1) Melody/tune; 2) Harmony; 3) Differences between loud and soft; 4) Different instruments/voices; 5) Lyrics in sung music*.* |
| 7 | I can hum or sing to the music I hear in my head whilst I am hearing it |
| 8 | I can hum or sing the music in my head after I have heard it |
| 9 | My body moves to the music I hear in my head (e.g., tapping a foot) |
| 10 | The music I hear in my head is familiar to me* |
| 11 | The music I hear in my head is my own creation |
| 12 | The music I hear in my head can change the mood I am in |
| 13 | The music I hear in my head makes me feel anxious |
| 14 | The music I hear in my head makes me feel excited |
| 15 | The music I hear in my head contributes to me feeling down and depressed |
| 16 | The music I hear in my head is pleasant |
| 17 | I can calm myself down by focusing on the music I hear in my head |
| 18 | The music I hear in my head reflects how I am feeling inside* |
| 19 | The music I hear in my head affects my relationships with other people* |
| 20 | I hear the music in my head in full (from start to finish), as melody with harmony |
| 21 | My inner music is shortened/condensed compared to normal, out-loud music |
| 22 | I hear the same music repeating itself in my head |
| 23 | I can control the music I hear in my head* |
| 24 | I know when I am about to have a music-hearing experience |
| 25 | I know what triggers my music-hearing experiences* 1) Yes; 2) No |
| 26 | I have mistaken the music playing in my head for real music playing externally* |
| 27 | If you have mistaken music in your head for external music |
|  | 1. When did this first occur?** |
|  | 1. Where did it feel like it was coming from?** |
|  | 1. Has this experience changed over time? 1) Yes; 2) No |
| 28 | How, if at all, are your music-hearing experiences different from hearing on a recording or played live?** |
| 29 | How, if at all, are your music-hearing experiences different from the process of remembering music?** |
| 30 | What other kinds of experience, if any, accompany your music-hearing experiences?** |
| 31 | Have you ever consulted your GP or a neurologist in relation to your music-hearing experiences? 1) Yes; 2) No |
| 32 | If there are any aspects of your music-hearing experiences that this questionnaire has not covered, please describe them here.** |
